# Civil Treatment of Accident Cases

**Published:** 1919-07-19

**Authors:** 


### WAR AND SURGICAL PROGRESS.

### IV.—Civil Treatment of Accident Cases.

THE marked improvement in results obtained in casualty clearing stations during the last two years of the war must have been very obvious to all who worked with these units, and if we consider mainly the cases of badly injured patients, the lessons derived from this experience should be found possible of application to many of the accidents and urgent surgical conditions which daily gain admission to our civil hospitals and nursing homes. In previous articles we have dealt with a few of the purely surgical aspects of these improvements and in the present sketch one would refer to an advance which has certainly gone a long way towards producing such creditable results, namely, the treatment of shock and allied conditions.

When it was realised that, except where internal hæmorrhage was suspected, it was definitely more advantageous to improve the general condition of the patient instead of rushing him at the earliest possible moment into the operating-theatre, the practice of deliberate resuscitation became more or less general and patients were frequently given even two or more hours of preparatory treatment before active surgical measures were applied.

### PRELIMINARY TREATMENT.

The first necessity is, however, to stop all external bleeding, and as it frequently happens that either time or circumstances prevents complete control by forceps or sutures locally, use may at once be made of a tourniquet and this left in position, according to most reports, for a very considerable time without any prejudice to ultimate recovery.

Next come the three essentials, warmth, rest, and fluid. For the first, a number of simple methods, such as wrapping in warmed blankets with containers of hot water, were found as effective as more complicated electrical and hot vapour devices. For the second, small doses of morphia combined with elemental principles in position and splinting were sufficient, but the satisfactory provision of extra fluid was to be found a more complex problem, and there is still some disagreement as to the best method.

Marshall states that, in his own experience and judging by observations on blood concentration and blood volume, the best results are obtained when fluids are given by the month. Also that next to this routine comes rectal infusion, since subcutaneous salines do not appear to benefit a case of very low blood pressure, remaining long unabsorbed. Of gum saline, he says that though no harm has been seen to result from this solution, as it sometimes undoubtedly does with saline, yet he has never obtained the strikingly good results reported by some observers.

The most important and probably by far the most valuable method for severe cases is whole blood transfusion. The subject of compatibility of blood is beyond the scope of these notes, but one may point out that the determination is simple in practice and also that instances of dangerous incompatibility are sufficiently rare to make it not worth while to create unnecessary delay by testing the bloods when dealing with desperate cases. In resuscitation work, with both surgical and medical conditions, where the patient's state is bad in the extreme, and when all other methods have failed, one has seen some remarkable recoveries following the injection of whole blood by means of the modified Kimpton tubes. The good effect is produced with extraordinary rapidity, and one can safely say that blood transfusion is a measure come to stay.

### THE OPERATION PERIOD.

The practical points in accessory treatment during actual surgical procedures may be conveniently grouped under two main heads: the eternal condition of patient's body and the anæsthetic employed. Body temperature must at all costs be maintained while he is in the theatre. Among the means available are employment of heated tables, efficiently heated theatres, and technique which provides the minimum exposure of body surface. Particularly should the patient be handled gently after operation. It is stated that sudden change in relative position of the trunk such as rolling over on the side or tilting up in lifting on to the trolley may produce a fall of blood-pressure followed by disastrous results later. Also, as formerly recognised, operative manipulation and exposure must be kept down; the surgeon can do much to reduce the shock of an operation when he realises that exposure of more than one coil of gut at a time outside the abdominal cavity will produce a marked and rapid pressure decrease. A certain revival of Criles' local infiltration methods of nerve-blocking at site of operation has occurred, but although this procedure has undoubtedly been most successful in practised hands, one must reserve judgment until its application has become more common in civil work.

### THE FUTURE CASUALTY DEPARTMENT.

The most urgent points for the consideration of those responsible for organising and equipping of casualty departments in our home hospitals are probably, first, that there should be adequate heating arrangements, electric or otherwise, not only in the wards and theatres but also in the receiving room; second, that modern splints should be immediately at hand to produce extension and fixation of fractures at the earliest possible moment; and third, that the out-patient department should be provided with every means of resuscitation treatment so that the customary "delay before admission" may actually be turned to the patient's advantage. Also we must remember that our younger generation, the newly qualified men upon

☛ Previous articles appeared on May 24, June 7 and 28, p. 319.

**War and Surgical Progress**—(*continued*).

whom considerable responsibility falls in being often the first to deal with severe casualty cases, should be made thoroughly cognisant of the lessons which war has taught us. Text-books are rapidly coming up abreast of the times and will undoubtedly take up the modern attitude, but such changes are slow, and at a period of such fast-moving reconstruction every care should be taken that these men do not learn and then have to unlearn the accepted methods of six years ago. The same principle applies in fact throughout the whole course of an accident case.

After Treatment.

A short time ago we gave a brief sketch of advances in anæsthetic administration, and the reduction of shock and other advantages produced by employment of the gas and oxygen method was then explained. Vomiting seldom follows in cases so treated, and one thus obtained the inestimable advantage of giving fluids by the mouth within a few minutes of operation. For the former custom of injecting strychnine, ether, camphor, pituitrin, etc., into patients whose post-operative condition was unsatisfactory, modern opinion can best be gauged by noting the frequency with which Crile's criticism has been cited by writers in the surgical journals. Likening the effect of those substances to that of a thumb-screw, Criles suggests that, "if we feel bound to do something to these cases, let us apply the thrumb-screw, it will be more economical!"

Otherwise our late means are exactly similar to the early. Warmth, rest and fluids are the essentials and provided by means already suggested, but here one may call attention to the use of morphia in relatively high doses. This was frequently done in the past with the customary objects in view, but many workers now point out that in shocked but restless cases an initial dose may produce no apparent effect, a second may be given with apparent benefit, but if resuscitation measures be successful and the general condition of the patient begin to improve, the morphia may take increased and dangerous effect, defeating all efforts towards further shock recovery.

Similarly might one collect an ever-increasing list of lessons, large and small, which war experiences have taught us, but the chief and all-embracing principle lies in fuller co-operation between surgeon, radiographer, anæsthetist, bacteriologist, and, in fact, every hospital department concerned with a surgical case. It is not only in research that we need "team work." In our home hospitals we have always some co-ordination of departments, but too frequently it is more official than practical in nature. On every hand one hears of proposals and schemes aiming towards the ideal, and these signs that the urgent necessity of reorganisation is at least realised must be more than gratifying to every progressive surgeon in the country.

